# Can eculizumab be discontinued in aHUS? Case report and review of the literature

**DOI:** 10.1097/MD.0000000000004330

**Published:** 2016-08-07

**Authors:** Tuncay Sahutoglu, Taner Basturk, Tamer Sakaci, Yener Koc, Elbis Ahbap, Mustafa Sevinc, Ekrem Kara, Cuneyt Akgol, Feyza Bayraktar Caglayan, Abdulkadir Unsal, Mohamed R. Daha

**Affiliations:** a Department of Nephrology, Sisli Hamidiye Etfal Educational and Research Hospital, Istanbul; b Department of Internal Medicine, Recep Tayyip Erdogan University, Rize, Turkey; c Department of Nephrology, C3-P, Leiden University Medical Center, Leiden, The Netherlands.

**Keywords:** aHUS, eculizumab, mutation of complement factors

## Abstract

**Background:**

The management of atypical hemolytic uremic syndrome (aHUS) has evolved into better control of thrombotic microangiopathy (TMA) and recovery of renal functions since the recent introduction of the terminal complement cascade blocker, eculizumab, into clinical use. Better characterization of genotype–phenotype relations has become possible with genetic and clinical studies. However, these advances brought up some important issues, such as the possibility and timing of discontinuation of eculizumab and strategy of follow-up that need to be enlightened.

**Case Summary:**

One of our aHUS cases with a novel complement factor H mutation, who developed unusual laboratory findings (thrombocytopenia and mild creatinine elevation without other features of TMA) following discontinuation of eculizumab was presented. Literature and case reports relevant to discontinuation of eculizumab in aHUS patients were reviewed.

**Conclusion:**

Limited experience suggests that the risk of recurrence of TMA following discontinuation of eculizumab is relatively low for patients with MCP mutations, homozygous CFHR3/R1 deletions, anti-CFH antibodies, CFI mutations, and no identifiable mutations, whereas there is a major risk for patients with CFH mutations. Early detection of TMA recurrence and prompt retreatment with eculizumab seem to be efficient in controlling of TMA and restoration of kidney functions.

## 1 Introduction

Hemolytic uremic syndrome (HUS) is a systemic microvascular disease, with thrombotic microangiopathy (TMA) is its fundamental histologic pathology and kidney injury is the most prominent clinical finding.^[1]^ Recent advances in understanding of the pathophysiology of thrombotic microangiopathy and laboratory tests have eased the differential diagnosis between the 4 groups of causes of this entity, namely thrombotic thrombocytopenic purpura, secondary HUS, shigella toxin-related HUS, and atypical HUS (complement mediated HUS, aHUS).^[2]^ Discovery of the distinct role of complement dysregulation as the primary cause of aHUS and the success of treatment of aHUS with terminal complement cascade blocker eculizumab have opened a new era in the management of this debilitating disease.^[3,4]^ Indeed, treatment of patients on long-term plasma therapy and progressive aHUS patients with eculizumab resulted in remission of TMA in majority of cases, clinically significant gain in glomerular filtration rate (GFR), and even recovery from dialysis.^[5]^ However, the episodic nature of the disease, exceptional cost, and potential side effects of eculizumab have raised a new and yet to be cleared questions. How long should eculizumab be continued once TMA has been taken under control and glomerular filtration rate has been stabilized? How should the strategy of follow-up of TMA be done while off treatment?

We herein present a patient with aHUS who was treated with and discontinued eculizumab and review the literature relevant to the discontinuation of eculizumab in aHUS patients.

## 2 Case

A previously healthy, 33-year-old white female was presented with headache and fever for 3 days. She did not used to smoke or consume alcohol. She gave 3 live healthy births and 1 year ago bilateral leg swellings and high blood pressure were noticed close to her last delivery, but medical investigation was not performed and her symptoms disappeared soon after the delivery. Her mother succumbed to a sudden disease, which was characterized by acute renal and neurological injuries, but further information was not available.

On physical examination, she was good on appearance, and temperature, blood pressure, and pulse rate were 38°C, 160/100 mmHg, and 110 bpm, respectively. Bilateral minimal pretibial edema was noticed.

The laboratory tests were consistent with thrombotic microangiopathy and severe renal dysfunction (leukocytes 5800 cells/mm^3^, urea 255 mg/dL, creatinine 11.8 mg/dL, uric acid 8.7 mg/dL, Na 133 mEq/L, K 4.9 mEq/L, AST 43 U/L, ALT 105 U/L, LDH 1248 U/L, total bilirubin 0.03 mg/dL, CPK 37 U/L, C-reactive protein <3 mg/L, 2–3 leukocytes and 8–10 erythrocytes per high power field and 3+ proteinuria in urinalysis, 24 hours proteinuria 2.4 g, serum haptoglobin <10 mg/dL, Coomb tests negative, reticulocytes 3.68%, and 5% schistocytes per field in peripheral blood film). Plasma ADAMTS13 levels and activity were within the normal limits. Antinuclear antibody was negative, C3 level was 80 mg/dL (85–200), and C4 level was within the normal range. Left renal agenesis and enlarged right kidney (145 × 55 mm) were detected by urinary ultrasonography.

Genetic analysis revealed a novel mutation in exon 21 of complement factor H (CFH) (c.3454T>A; p.C1152S), and the same mutation was later identified in her asymptomatic 3 (males) of 4 siblings.

Daily plasma exchange using 40 mL/kg fresh frozen plasma and on-demand hemodialysis were started. Markers of thrombotic microangiopathy did not consistently normalize during 22 sessions of plasma exchange; therefore, PE was replaced by eculizumab within 2 weeks of vaccination against *Neisseria meningitides* (900 mg/week for 4 weeks, 1200 mg every other week from the 5th week on). Thrombocytopenia and elevated LDH normalized within 1 month along with gradual improvement in renal functions and the need for dialysis was eliminated within 2 months of eculizumab treatment (Fig. 1 A, B). Eculizumab was discontinued after 1 year of treatment, during which creatinine nadir was 1.35 mg/dL, and the patient was set to follow-up. Thrombocytes dropped and remained below the lower limit of normal from the 7th month (January 6, 2015) of follow-up on, but LDH levels remained around the upper limit of normal (Fig. 1 C). Multiple peripheral blood films, serum haptoglobin levels, and reticulocyte counts were found normal, except for thrombocytopenia, since detection of thrombocytopenia. Levels of creatinine slightly increased but remained <2 mg/dL except for a few occasions, whereas the levels of proteinuria remained <0.5 g/day (385 mg/day at last visit) (Fig. 1 D). Informed consent was obtained from the patient.

**Figure 1:**
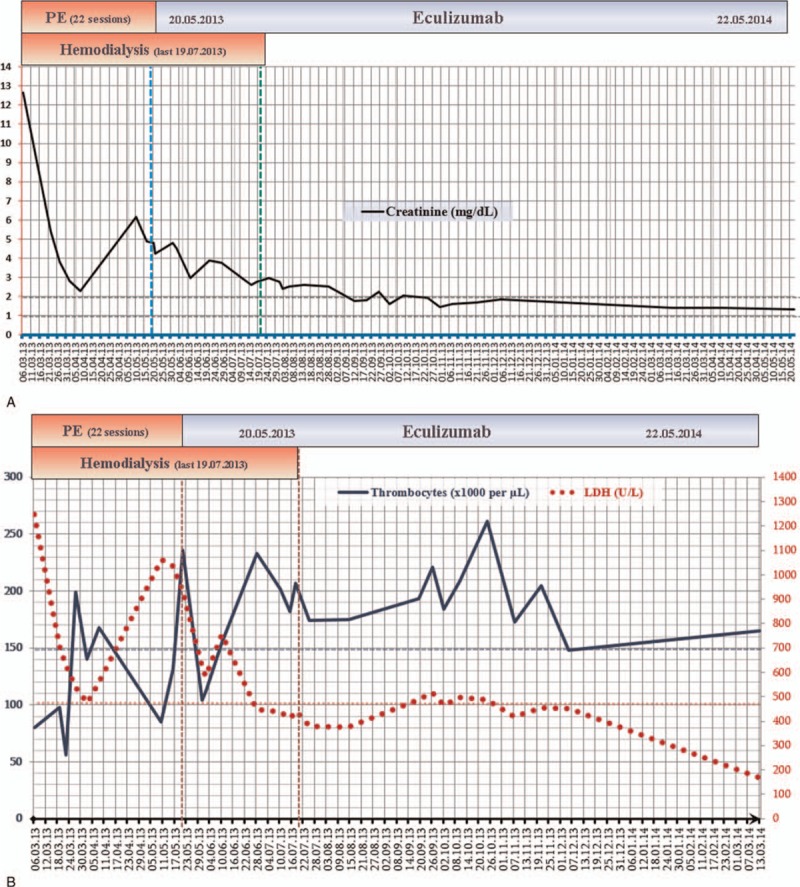
(A) Creatinine levels decrease initially with plasma exchange and hemodialysis, but rise again under plasma exchange treatment. Treatment with eculizumab induces steady decline in creatinine levels and later allows to discontinue hemodialysis. (B) Thrombocyte counts and lactate dehydrogenase (LDH) levels change initially toward normal ranges, but return to abnormal levels under plasma exchange and hemodialysis. Treatment with eculizumab results in consistent normalization of both thrombocyte counts and LDL levels. (C) The course of LDH levels and thrombocyte count during off treatment follow-up shows that thrombocyte counts drop and remain <150,000 cells/μL since the 7th month of discontinuation of eculizumab, whetreas LDH levels remain mostly just below the upper limit of normal. (D) Creatinine levels during off treatment follow-up swing around 1.6 mg/dL, which is 0.25 mg/dL higher than the nadir level of 1.35 mg/dL under eculizumab treatment.

**Figure 1 (Continued):**
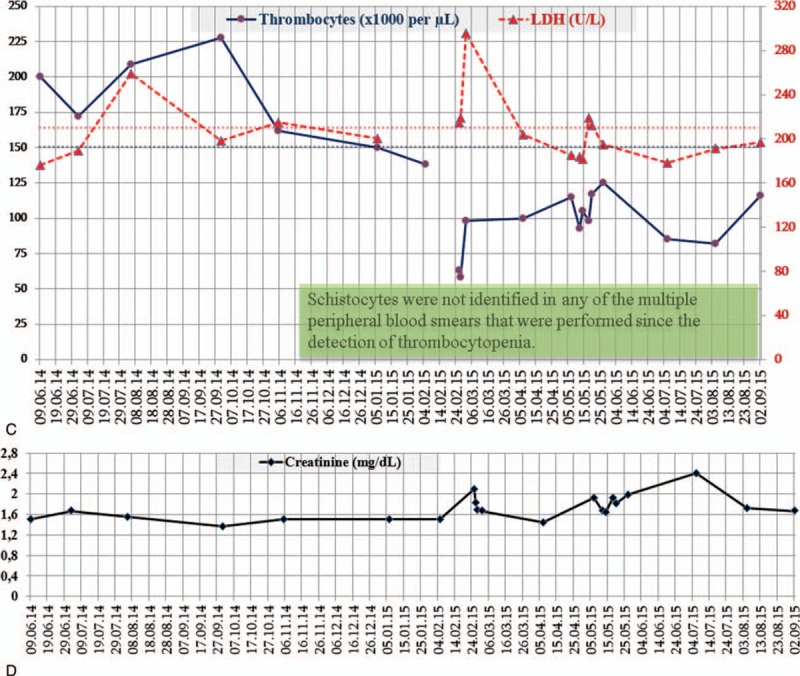
(A) Creatinine levels decrease initially with plasma exchange and hemodialysis, but rise again under plasma exchange treatment. Treatment with eculizumab induces steady decline in creatinine levels and later allows to discontinue hemodialysis. (B) Thrombocyte counts and lactate dehydrogenase (LDH) levels change initially toward normal ranges, but return to abnormal levels under plasma exchange and hemodialysis. Treatment with eculizumab results in consistent normalization of both thrombocyte counts and LDL levels. (C) The course of LDH levels and thrombocyte count during off treatment follow-up shows that thrombocyte counts drop and remain <150,000 cells/μL since the 7th month of discontinuation of eculizumab, whetreas LDH levels remain mostly just below the upper limit of normal. (D) Creatinine levels during off treatment follow-up swing around 1.6 mg/dL, which is 0.25 mg/dL higher than the nadir level of 1.35 mg/dL under eculizumab treatment.

## 3 Discussion

We have reported an aHUS case caused by CHF mutation and successfully treated with and discontinued eculizumab with an unusual course of follow-up. The outcome of patients who discontinue eculizumab treatment used to be either stable or relapse of TMA mostly along with acute deterioration in renal functions.^[6]^ The present case, however, developed only thrombocytopenia and mild increase in creatinine levels above nadir (from 1.35 mg/dL or 51 mL/min/1.73m^2^ to 1.65 mg/dL or 40 mL/min/1.73 m^2^ at the last visit), whereas the proteinuria remained <0.5 g/day. These features may appear like insignificantly faint at first glance, but it should be noticed that the estimated GFR decreased by 11 mL/min/1.73m^2^ in 16 months of follow-up. Kidney biopsy could add valuable inputs for further characterization of these findings, but solitary kidney presented a relative contraindication. Factors that have been associated with thrombocytopenia were sought, but none was identified. Therefore, we concluded that thrombocytopenia could either be due to subclinical “smoldering” aHUS or immune thrombocytopenia. Retreatment with eculizumab could be tried and interesting findings could be derived regarding its effects on slightly increased creatinine levels and thrombocytopenia, but the absence of other components of TMA and the excessive price of the drug lead to disapproval by the insurance system.

The efficacy of eculizumab in the treatment of aHUS has been shown in a number of case reports and in the phase 2 study reported by Legendre et al.^[5]^ But once satisfactory aims of a treatment have been reached, new goals dawn on the horizon, and safe discontinuation of eculizumab is probably one of the most important issues today in aHUS. This is because aHUS is characterized by episodic attacks of disease that is thought to represent an environmental trigger upon a genetically suitable patient.^[3]^ Asymptomatic relatives with the exact mutations in complement pathway genes and TMA-free periods interspersed between episodes of disease attacks in aHUS patients are supportive of this concept. These findings suggest that there might be some periods of time where complement inhibition is unnecessary. However, randomized controlled trials on the safety and outcomes of discontinuation of eculizumab have not been published yet, but the phase 2 study of eculizumab in aHUS, case reports/series, and bench studies could provide with some insights. The 2-year extension of phase 2 eculizumab study revealed that hematological normalization, which was one of the primary end points, was achieved in 76%/90%, 88%/90%, and 88%/90% of patients, whereas gains in GFR were +33/+6, +25/+7, and +37/+8 mL/min/1.73 m^2^ in acute progressive and chronic TMA patients within 26 weeks, 1 year, and 2 years of eculizumab treatment, respectively.^[7]^ The differences in changes of GFR between 26 weeks and 1 or 2 years of eculizumab treatment were not statistically significant in both of the subgroups.^[7]^ These results suggest that there is little benefit if any in terms of renal functions with extending the duration of eculizumab treatment after TMA has resolved and creatinine levels have been stabilized, but it also should be kept in mind that all patients, except for 2, had GFR <60 mL/min/1.73 m^2^ at study entry; therefore, GFR changes may need to be interpreted according to chronic kidney disease.

The underlying genetic mutations have been shown as important indicators of clinical outcomes and recurrence rates, with complement factor H, I, and C3 abnormalities having the highest rates of recurrence after transplantation.^[1,8]^ However, these reports belong to the time before widespread availability of eculizumab in the treatment of aHUS; therefore, relapse rates, strategy of follow-up, and outcomes after attempted withdrawal of eculizumab have not been clarified. The study by Legendre et al provided limited data about discontinuation of eculizumab, that was one of 4 patients developed TMA on the 80^th^ day and retreatment with eculizumab resulted in control of hemolysis with gradual renal improvement.^[7]^ Therefore, we aimed to analyze the outcome of patients who discontinued eculizumab in more details and 7 studies with a total of 24 cases were identified that can be reviewed in this regard (Table 1). In this analysis, eculizumab was restarted in all cases 8 patients (33%) who relapsed within an average of 4.5 months (range 0.7–17.3 months) of follow-up since its discontinuation and 310.8 patient-months of drug-free follow-up were gained in return. Among them, 1 patient with no identified mutation lost her limitedly functioning (creatinine 1.8–2 mg/dL before discontinuation) second kidney transplant, 1 patient had unreported outcome data, and 6 patients had favorable outcomes (all patients were dialysis-free, reported creatinine levels of 5 patients remained below or nearly equal to the levels before discontinuation of eculizumab). The overall relapse rates in patients with combined complement abnormalities were 100% in the presence of CFH mutation and 50% in homozygous deletions of CFHR3/R1 plus anti-CFH antibodies. The frequencies of relapses in patients with isolated complement abnormalities were 60% for CFH mutation, 25% for CFI, and 0% for MCP, homozygous CFHR3/R1 deletions, and anti-CFH antibodies. When the Table 1 was analyzed in further details according to the underlying complement abnormalities, the following findings were revealed.

**Table 1:**
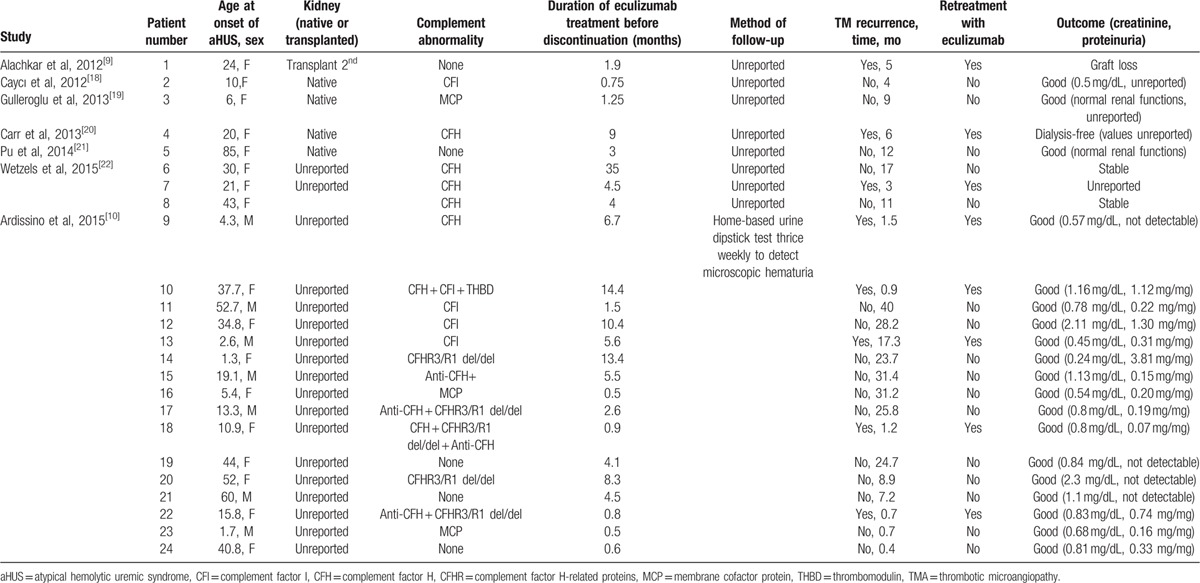
Summary of aHUS cases who were treated with and discontinued eculizumab.

Relapse was reported in 1 of 5 patients (20%) without detected complement abnormalities (patient 1) after 5 months of discontinuation of eculizumab with a consequence of graft lost, whereas the other 4 patients (patients 5, 19, 21, 24)who were being followed-up for a mean of 11 months (0.4–24.7) after drug discontinuation had favorable outcomes with no relapse. CFH mutation was reported in 7 patients (patients 4, 6, 7, 8, 9, 10, 18) and 5 of them (71%) (3 of the 5 with isolated CFH mutations and 2 of the 2 with combined complement abnormalities) relapsed within a mean of 2.5 months (0.9–6) after discontinuation of eculizumab; the outcomes of 3 patients who were reported by Ardissino et al were good, whereas 1 patient remained free of dialysis (patient 4) and the outcome of the other patient (patient 7) was not reported. CFI mutation was reported in 5 (patients 2, 10, 11, 12, 13) patients and relapse was detected in 2 of them (1 of 1 with combined and 1 of the 4 with isolated complement abnormalities; patients 10 and 13, respectively) after 0.9 (patient 10) and 17.3 (patient 13) months of eculizumab discontinuation; the outcomes were good in both. The outcomes of 3 patients (all with isolated complement abnormalities, patients 3, 16, 23) with MCP mutations were good and no relapse was reported after a mean of 13.6 months (0.7–31.2) of eculizumab discontinuation. The only patient (patient 10) who carried THBD mutation in combination with CFH and CFI mutations relapsed after 0.9 month of eculizumab discontinuation with a good outcome. Homozygous deletions of CFHR3/R1 were reported in 5 patients (patients 14, 17, 18, 20, 22) and 2 of them (patients 18, 22) relapsed (2 of 3 patients with combined complement abnormalities) after 1.2 and 0.7 months of eculizumab discontinuations and their outcomes were good. Anti-CFH antibodies were detected in 4 patients (patients 15, 17, 18, 22) and relapse was reported in 2 cases (2 of the 3 with combined complement abnormalities, patients 18 and 22) after 1.2 and 0.7 months of eculizumab discontinuations with good outcomes in both.

The only case who had permanent renal damage following relapse has some important features to be pointed out.^[9]^ Her risk of recurrence could be foreseen very high as she had already lost her native and 1st transplanted kidney functions under plasma exchange therapy after the initial episode and first recurrence of TMA, respectively. Furthermore, the second TMA relapse, which occurred 3.5 months after her 2nd kidney transplantation, was initially treated with plasma exchange and eculizumab was added later, after which partial renal recovery and complete remission of TMA could be obtained. The 3rd relapse, which occurred 5 months following discontinuation of eculizumab, was triggered by pneumonia and intervening endovascular procedure caused further renal injury while renal recovery was ongoing. In comparison, home-based urine dipstick test 3 times a week was incorporated into routine follow-up and permanent damage was not reported in any case by Ardissino et al despite the fact that significant acute renal injury was present in all 3 of their relapsed patients as they were presented in a previous report.^[10,6]^ Favorable outcomes of the latter study might be because of early detection of relapse and restarting of eculizumab. However, there is not any established strategy yet to be adopted in this regard, but according to Ardissino et al,^[6]^ home-based urine dipstick test was able to identify all of the 3 cases who relapsed with only 2 false positive results. Although not validated, it is an easy and cheap test that is probably worth to be performed, which could save a few days/weeks before the development symptoms or date of next outpatient appointment. However, urine dipstick test and other fundamental laboratory indicators of TMA can only be detected after significant endothelial or renal injury has occurred, which is not ideal. A recent study by Noris et al showed that an in vitro human microvascular endothelial cell model could indicate complement activation in the fluid phase and adequacy of complement inhibition, which might be capable of predicting pending disease flare up.^[11]^ If such a model can be validated, standardized, and made widely accessible/affordable, it could reveal the status of complement activity, which can be used to evaluate the adequacy of complement inhibition and timing of drug discontinuation and reinstitution.

The paucity of available evidence precludes making strong suggestions regarding the strategy of discontinuation of eculizumab. Furthermore, the types of mutations in each culprit complement factor are not uniform, and as different mutations in a single gene can result in different genotype–phenotype consequences, one should anticipate that carriers of different types of mutation in a single gene could have different clinical phenotypes.^[3]^ However, the aforementioned experiences suggest that discontinuation of eculizumab and close follow-up in patients with isolated MCP mutations, homozygous CFHR3/R1 deletions, or anti-CFH antibodies seem to be safe once TMA has resolved and renal functions have been stabilized. Patients with no identifiable complement abnormality, CFI mutations, or homozygous CFHR3/R1 deletions plus anti-CFH antibodies are under considerable risk of recurrence. CFH mutations in isolation or in combination with other complement abnormalities have a very high risk of recurrence and patients with limited renal functions should probably have lifelong eculizumab treatment, whereas the termination of treatment in cases with normal renal functions should be left on the discretion of physicians and patients. The treatment of patients with anti-CFH antibodies presents significant differences than others. Anti-CFH antibodies have been shown to be pathogenic and thought to develop because of homozygous deletions of CFHR3/R1 proteins in majority of cases.^[12]^ A combination of plasma exchange plus immunosuppressive regimen has been shown to induce and maintain remission with low serum anti-CFH antibody levels.^[13,14,15,16]^ Therefore, it suggests that after low titers of anti-CFH antibodies have been attained, discontinuation of eculizumab can be considered if it is used, and the monitoring of anti-CFH antibody titters should be included in the follow-up if available. Experience about discontinuation of eculizumab in patients with CFB and C3 mutations has not been published, but these 2 mutations lead to end-stage renal disease in about 60% to 70% of patients and recurrence risk after kidney transplantation was found significantly high.^[1,17]^ Therefore, approach to patients with these mutations should be similar to those with CFH mutations.

## 4 Conclusions

Evidence to guide the discontinuation of eculizumab in patients with aHUS is weak. However, limited experience suggests that patients with MCP mutations, homozygous CFHR3/R1 deletions, anti-CFH antibodies, no identifiable mutations and CFI mutations carry a low risk, whereas CFH mutations pose a major risk of recurrence of TMA following discontinuation of eculizumab in patients with aHUS. Preinjury markers of complement/TMA activation are needed, as early detection of TMA recurrence and prompt retreatment with eculizumab seem to be efficient in controlling of TMA and restoration of normal renal functions. Therefore, the decision on discontinuation of eculizumab should be customized individually for patients.
